# Second Wave, Late-Stage Neuroinflammation in Cleared Brains of Aged 5xFAD Alzheimer’s Mice Detected by Macrolaser Light Sheet Microscopy Imaging

**DOI:** 10.3390/ijms242317058

**Published:** 2023-12-02

**Authors:** Suk Hyun Lee, Hye Joo Son

**Affiliations:** 1 Department of Radiology, Hallym University Kangnam Sacred Heart Hospital, Hallym University College of Medicine, Seoul 07441, Republic of Korea; 2 Department of Nuclear Medicine, Dankook University Medical Center, Dankook University College of Medicine, Cheonan 31116, Republic of Korea

**Keywords:** Alzheimer’s disease, late-phase neuroinflammation, astrogliosis, 5xFAD, light-sheet microscopy, tissue clearing

## Abstract

This study leverages the innovative imaging capabilities of macrolaser light-sheet microscopy to elucidate the 3D spatial visualization of AD-associated neuropathologic networks in the transparent brains of 44-week-old 5xFAD mice. Brain samples from ten AD and seven control mice were prepared through a hydrophilic tissue-clearing pipeline and immunostained with thioflavin S (β-amyloid), anti-CD11b antibody (microglia), and anti-ACSA-2 antibody (astrocytes). The 5xFAD group exhibited significantly higher average total surface volumes of β-amyloid accumulation than the control group (AD, 898,634,368 µm^3^ [383,355,488–1,324,986,752]; control, 33,320,178 µm^3^ [11,156,785–65,390,988], *p* = 0.0006). Within the AD group, there was significant interindividual and interindividual variability concerning the number and surface volume of individual amyloid particles throughout the entire brain. In the context of neuroinflammation, the 5xFAD group showed significantly higher average total surface volumes of anti-ACSA-2-labeled astrocytes (AD, 59,064,360 µm^3^ [27,815,500–222,619,280]; control, 20,272,722 µm^3^ [9,317,288–27,223,352], *p* = 0.0047) and anti-CD11b labeled microglia (AD, 51,210,100 µm^3^ [15,309,118–135,532,144]; control, 23,461,593 µm^3^ [14,499,170–27,924,110], *p* = 0.0162) than the control group. Contrary to the long-standing finding that early-stage neuroinflammation precedes the subsequent later-stage of neurodegeneration, our data reveal that the second wave, late-stage active neuroinflammation persists in the aged AD brains, even as they continue to show signs of ongoing neurodegeneration and significant amyloid accumulation.

## 1. Introduction

Affecting upward of 10% of individuals older than 65 years, Alzheimer’s disease (AD) represents the most prevalent neurodegenerative disorder globally, leading to marked cognitive impairment and irreversible memory deficits [1,2]. While the current standard AD diagnostic paradigm largely hinges on neuropsychological evaluations, these approaches have their own set of limitations, including suboptimal accuracy in cognitive assessments. Insight into the natural course of the pathologic propagation of Alzheimer’s disease (AD) is essential for both delineating disease stages and guiding the timing for therapeutic interventions.

While the classic biomarker approach has focused on brain amyloid-beta positron emission tomography (PET) and cerebrospinal fluid (CSF) markers, these techniques are notably costly, invasive, and not broadly accessible for routine screening. Macrolaser light-sheet illuminating microscopy (Macro-LSFM), combined with tissue-clearing technologies, heralds a transformative paradigm in biomedical imaging, allowing in-depth 3D visualization of neuropathologic and cellular networks in a transparent intact mouse brain at high resolution [3,4]. First introduced to the scientific landscape in 2004, light-sheet fluorescence microscopy (LSFM) provides exceptional three-dimensional spatial resolution alongside high volumetric imaging speed, achieved by the deployment of a micrometer-thin laser beam to scan expansive fields in large biological specimens at cellular resolution [5,6]. Unlike conventional imaging methods that frequently require labor-intensive tissue sectioning and mounting, LSFM, integrated with tissue clearing, enables full-scale analysis of unsectioned specimens, thus preserving their architectural complexity while overcoming traditional limitations. This innovative tool paves the way for a detailed exploration of intact mouse brains and spinal cords at the cellular level, serving as a pivotal tool in understanding the extent of CNS deterioration in some animal models of neurodegenerative diseases in order to investigate the intricate interactions among amyloid or tau pathology and hundreds of different cell types forming intricate circuits and networks consisting of neurons, astrocytes, and microglia, which are extremely difficult to understand from 2D slices [7,8]. In this study, we leveraged the innovative imaging capabilities of Macro-LSFM with a hydrophilic tissue-clearing technique to elucidate the 3D spatial distribution of AD-associated neuroinflammation and neuropathologic changes in the brains of aged transgenic AD mice.

## 2. Results

**Figure 1:**
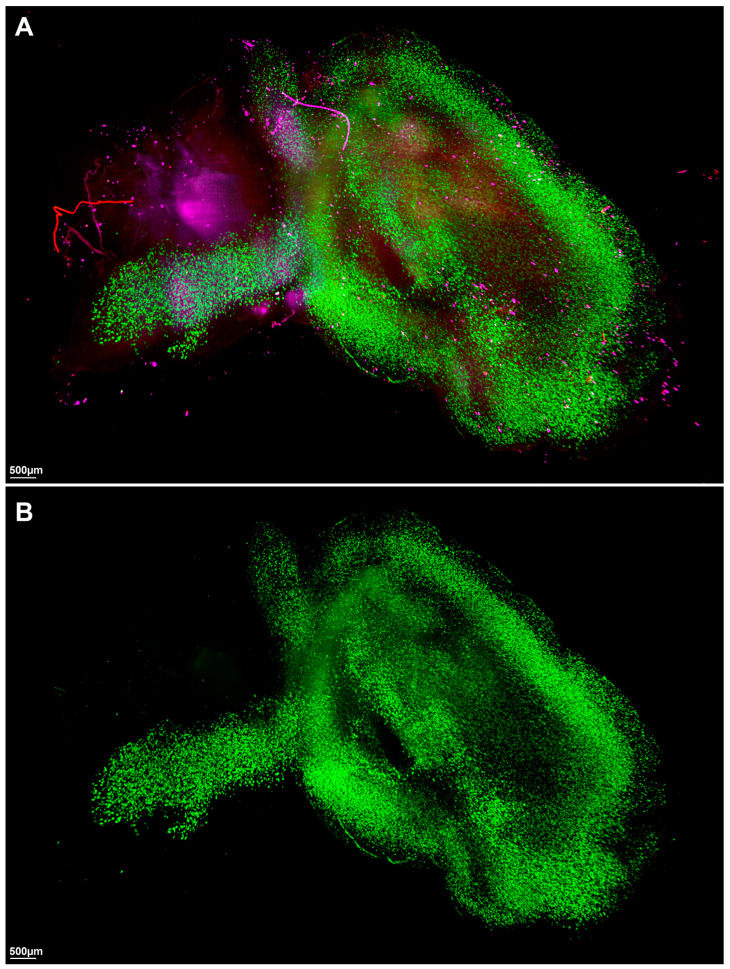

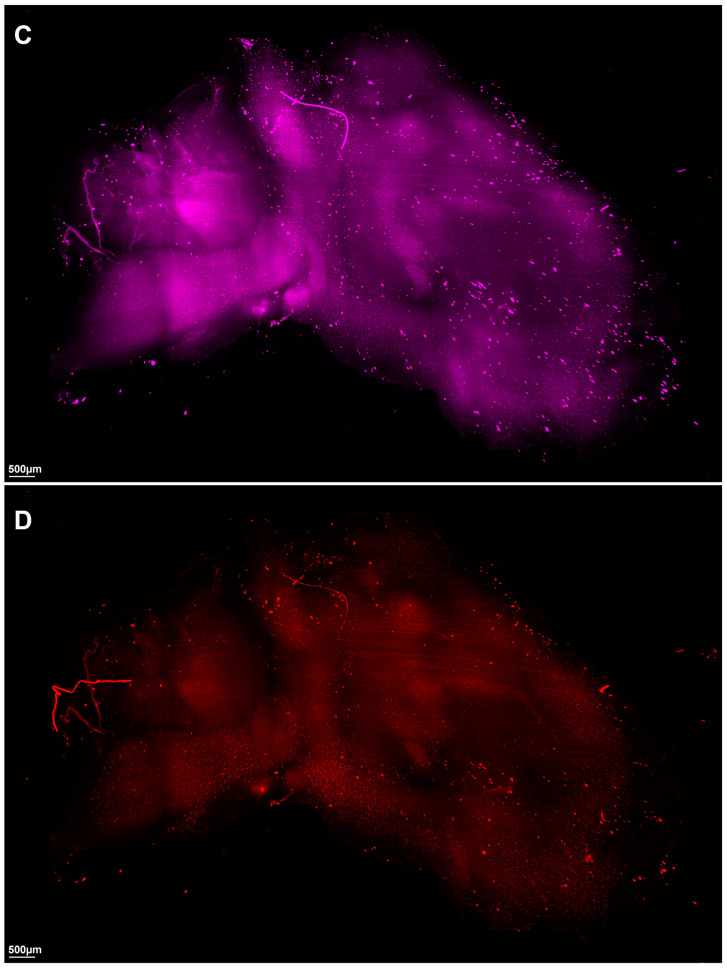
Visual overview of representative macrolaser light-sheet illuminating microscope imaging with hydrophilic tissue clearing and volume immunostaining of the brains of 44-week-old 5xFAD mice. (**A**) Merged image. (**B**) Green channel (488 nm) with volume immunostaining with Thioflavin S, a specific marker for amyloid. (**C**) Purple channel (647 nm) with volume immunostaining with anti-ACSA-2, a specific marker for astrocyte. (**D**) Red channel (561 nm) with volume immunostaining with an-ti-CD11b, a specific marker for microglia.

**Figure 2:**
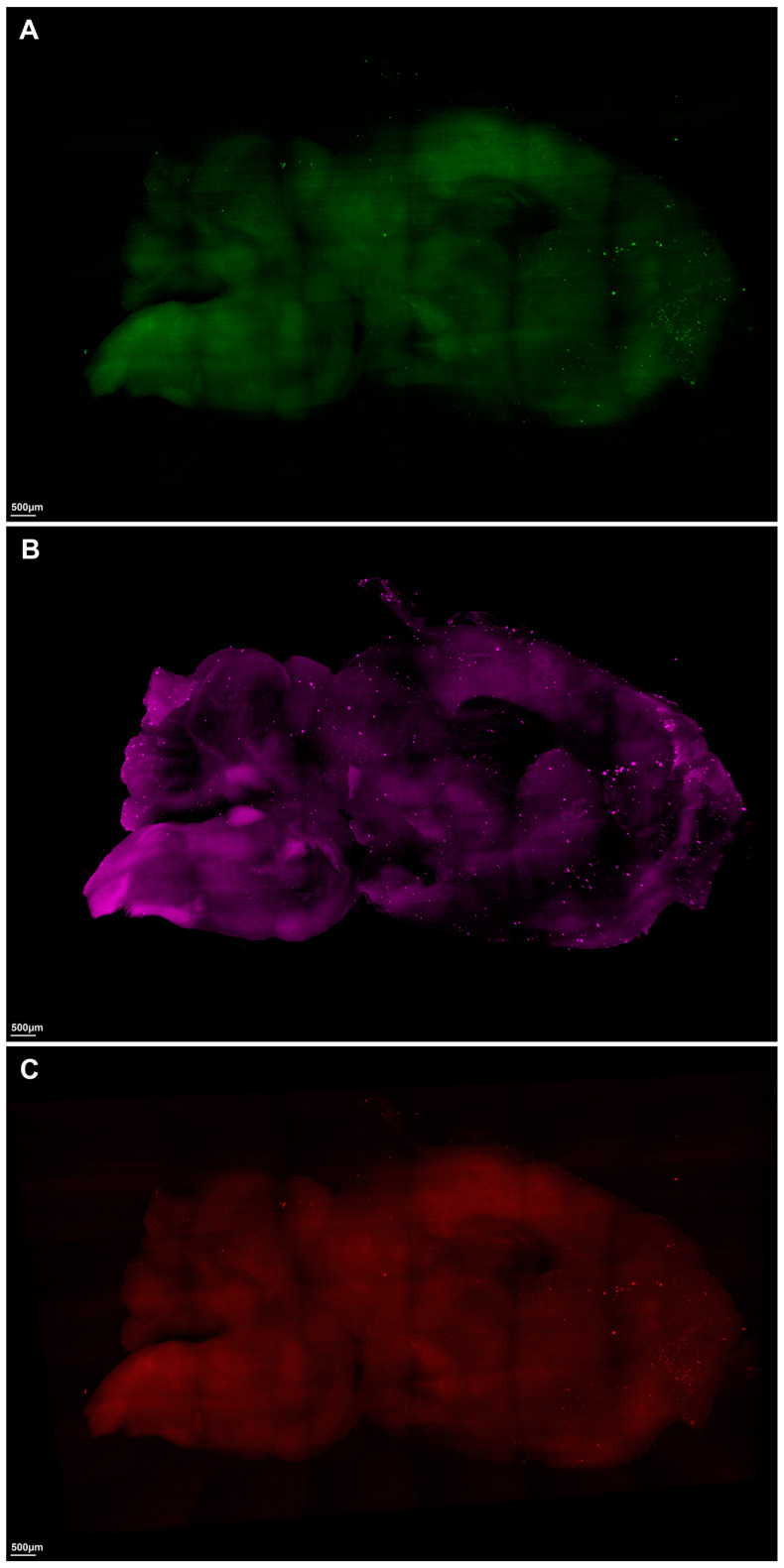
Visual overview of representative macrolaser light-sheet illuminating microscope imaging with hydrophilic tissue clearing and volume immunostaining of the brains of *C57BL* control mice. (**A**) Green channel (488 nm) with volume immunostaining with Thioflavin S, a specific marker for amyloid. (**B**) Purple channel (647 nm) with volume immunostaining with anti-ACSA-2, a specific marker for astrocyte. (**C**) Red channel (561 nm) with volume immunostaining with an-ti-CD11b, a specific marker for microglia.

Utilizing advanced macrolaser light-sheet microscopy in conjunction with state-of-the-art tissue-clearing chemical techniques, we conducted a comprehensive investigation into the three-dimensional spatial distribution of the key neuropathological markers thioflavin S for β-amyloid, anti-CD11b for microglia, and anti-ACSA-2 for astrocytes in the brains of 44-week-old 5xFAD mice, which are Alzheimer’s disease (AD) model mice, and *C57BL* control mice (Figure 1 and Figure 2, Supplemental Movie S1).

**Figure 3:**
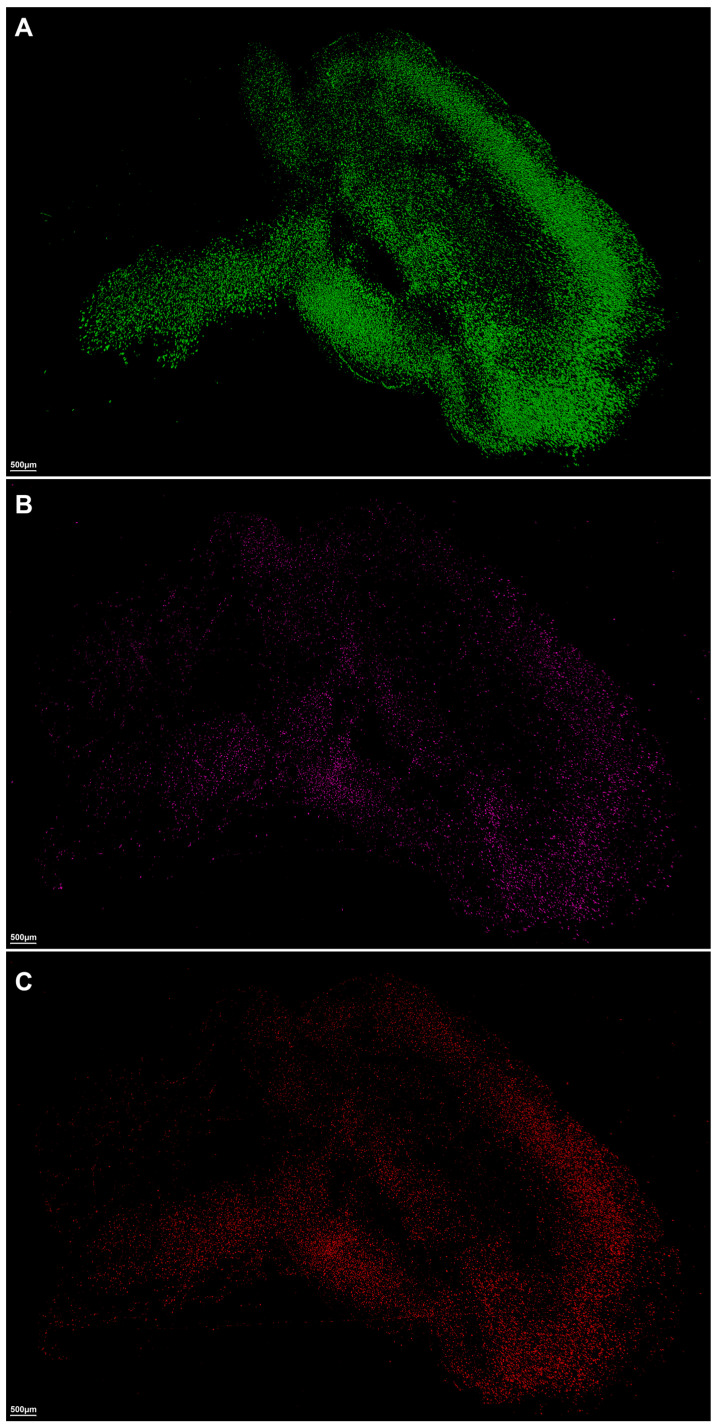
Visual overview of the Imaris imaging analysis software-based volume rendering surface model for amyloid, astrocyte, and microglia. (**A**) Total surface volume of Thioflavin S (Green channel: 488 nm) specific for amyloid. (**B**) Total surface volume of anti-ACSA-2 (Purple channel: 647 nm) specific for astrocytes. (**C**) Total surface volume of anti-CD11b (Red channel: 561 nm) specific for microglia.

**Figure 4:**
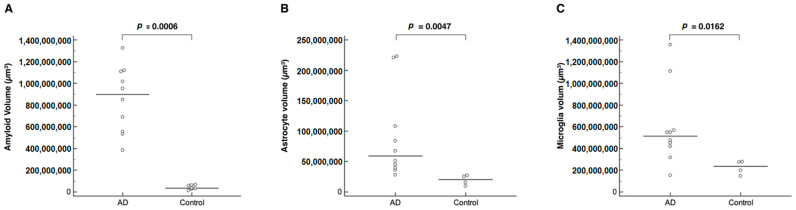
Comparison of the total surface volume of amyloid (**A**), astrocytes (**B**), and microglia (**C**) between the Alzheimer’s disease (AD, *n* = 10) and control mouse (*n* = 7) groups. The amyloid, astrocyte, and microglia volumes of the AD mouse group were significantly larger than those of the control mouse group and showed a diverse distribution.

Utilizing Imaris software (Version 7.2.3, Bitplane AG, Zurich, Switzerland) for advanced quantitative image analysis, we quantified the total surface volume of these three specific markers for β-amyloid, astrocytes, and microglia, revealing notable differences between the Alzheimer’s disease and control models (Figure 3 and Figure 4, Table 1). In the context of β-amyloid deposition in brain tissues, the 5xFAD group exhibited significantly higher average total surface volumes of β-amyloid accumulation compared to the control group (AD, 898,634,368 µm^3^ [383,355,488–1,324,986,752]; control, 33,320,178 µm^3^ [11,156,785–65,390,988], *p* = 0.0006) (Table 1, Figure 4A). 

**Figure 5:**
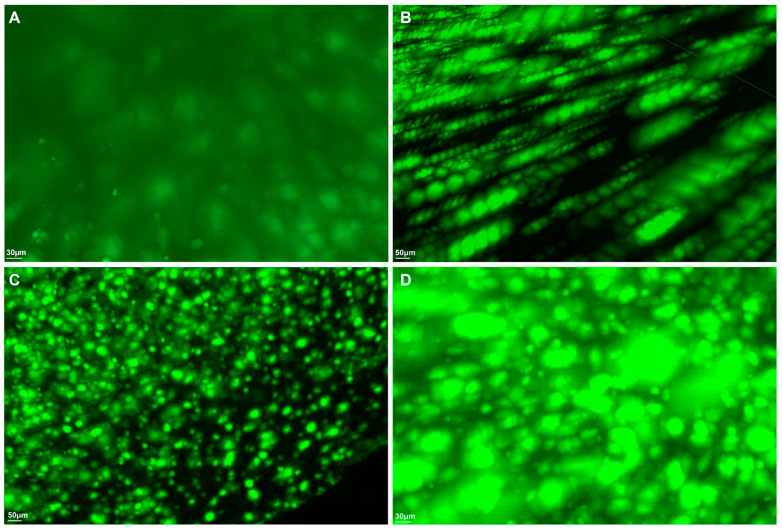
Intraindividual variability in 3D morphological features of amyloid particles within an Alzheimer brain. (**A**) diffuse plaque, (**B**) protofibrils, (**C**) small-sized, dense-core monomers or dimers, (**D**) high-weight oligomers.

**Figure 6:**
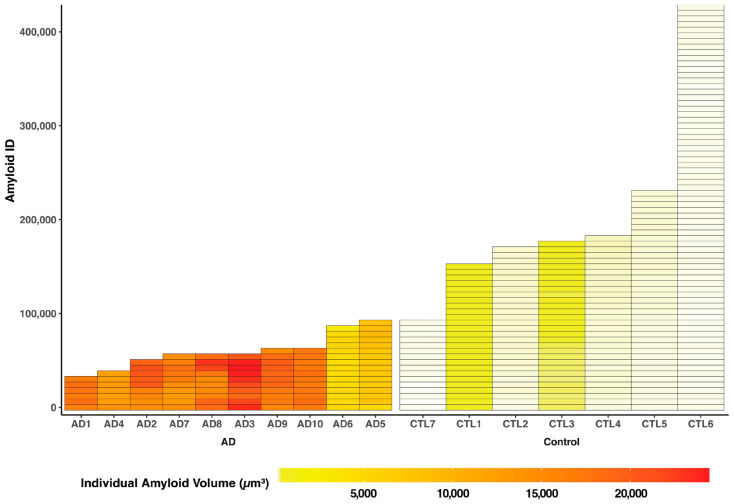
Intraindividual and Interindividual variability concerning the number and surface volume of each individual amyloid particle throughout the entire brain architecture among mice (total *n* = 17, AD = 10, control = 7). The X-axis corresponds to distinct mouse IDs in both the AD and control groups. The Y-axis indicates the unique IDs of individual amyloid particles within each mouse’s brain, while the Z-axis quantifies the volume of individual amyloid particles (µm^3^).

**Figure 7:**
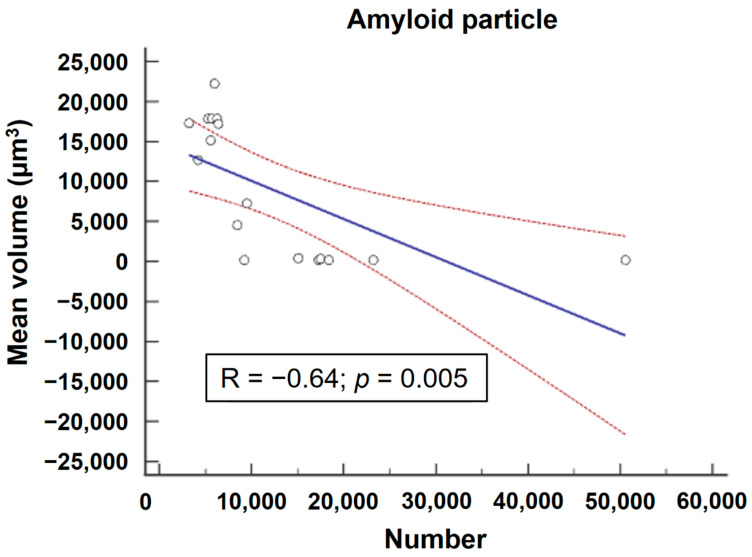
Correlation between the number and mean volume of amyloid particles (*n* = 17).

In each AD brain specimen, a heterogeneous array of amyloid particles manifesting diverse morphological profiles, including various sizes, shapes, and volumetric characteristics, was evident (Figure 5). Within our imaging dataset, we predominantly identified the presence of protofibrils (Figure 5B); small-sized, dense-core monomers (Figure 5C); and high-weight oligomers (Figure 5D) dispersed across the specimens, alongside localized occurrences of diffuse plaques (Figure 5A). Within the AD group, there was significant intraindividual and interindividual variability concerning the number and surface volume of each individual amyloid particle throughout the entire brain architecture (Figure 6, Supplemental Movie S2). There was a significant strong inverse correlation between the mean volume and the number of amyloid particles (R = −0.64, *p* = 0.0054) (Figure 7).

In the context of neuroinflammation, a significantly elevated expression of both astrocyte and microglia markers was conspicuously observed in the brains of 44-week-old 5xFAD mice compared to the negligible expression in the control mice (Figure 1, Supplemental Movie S1). Specifically, the 5xFAD group showed significantly higher average total surface volumes of anti-ACSA-2-labeled astrocytes than the control group (AD, 59,064,360 µm^3^ [27,815,500–222,619,280]; control, 20,272,722 µm^3^ [9,317,288–27,223,352], *p* = 0.0047) (Table 1, Figure 4B). Similarly, we observed a marked upregulation of microglial activation, as evidenced by anti-CD11b labeling, in the 5xFAD group relative to the control group (AD, 51,210,100 µm^3^ [15,309,118–135,532,144]; control, 23,461,593 µm^3^ [14,499,170–27,924,110], *p* = 0.0162) (Table 1, Figure 4C). 

## 3. Discussion

Herein, using macrolaser light-sheet illuminating microscopy (Macro-LSFM) with hydrophilic tissue-clearing technologies allowing 3D spatial visualization of AD-associated cellular and neuropathologic networks in transparent intact mouse brains, our results revealed that active neuroinflammation persists in the brains of 44-week-old 5xFAD mice, marked by substantial amyloid deposition, challenging the traditional sequence of early neuroinflammation leading to late-stage neurodegeneration. 

In 1902, the development of the “Ultramicroscope” by Richard Zsigmondy and Henry Siedentopf marked a seminal advance in biological imaging by introducing an innovative light-sheet microscope that diverged from traditional optical architectures through the separation of illumination and detection pathways, thereby establishing the first orthogonal light-sheet microscopy technique [9]. Nearly a century thereafter, in 2004, Macro light-sheet fluorescence microscopy (Macro-LSFM) emerged as a notable milestone, utilizing the orthogonal plane architecture to achieve optical sectioning of guinea pig cochlea [9]. Before the advent of Macro-LSFM, traditional imaging modalities presented several limitations that hindered their application in neuroscience contexts. Animal PET/CT systems, despite their unmatched capabilities in offering invaluable functional insights into metabolic and molecular processes, face several limitations that make Macro-LSFM a compelling alternative. The lower spatial resolution and extended acquisition time of PET/CT are eclipsed by the rapid and high-resolution imaging capabilities of Macro-LSFM [10]. Additionally, the ionizing radiation exposure inherent in PET/CT raises ethical and safety concerns in longitudinal animal studies [10]. Moreover, the requirement for specialized radiotracers in PET/CT limits its application to certain biological pathways and necessitates close proximity to a cyclotron, in contrast to the more versatile and easily administered fluorescent markers used in Macro-LSFM [10]. PET/CT lacks the high-speed optical sectioning capabilities of Macro-LSFM, making the latter a more suitable choice for capturing intricate three-dimensional cellular structures. In contrast, point-scanning imaging systems, such as confocal or two-photon microscopes, excel at three-dimensional optical sectioning and offer impressive spatial resolution. However, these point-scanning methods suffer from an intrinsic drawback: their sequential voxel-by-voxel scanning processes considerably bottleneck the temporal resolution, hampering the speed at which images can be captured [11]. This is particularly problematic in confocal microscopy, where the high light exposure of the specimen further limits long-term imaging capabilities and elevates the risk of phototoxicity and photobleaching [3]. While two-photon and multiphoton systems offer the advantage of greater depth penetration, they do so at the expense of complicated setup requirements, sluggish acquisition rates, and an elevated risk of photodamage. Conversely, traditional wide-field microscopes, capable of swift image acquisition via parallel signal collection from numerous volume elements, fall short in providing optical sectioning capabilities, thus making them less than ideal for three-dimensional imaging of thicker specimens [3]. Macro-LSFM, combined with tissue-clearing technologies, not only addresses but also overcomes many of the limitations inherent in traditional imaging modalities, marking a significant shift in the landscape of biomedical imaging. Offering an expansive whole field of view without compromising spatial resolution, this innovative system delivers rapid, high-resolution, exceptional in-depth three-dimensional visualizations of complex neuropathological and cellular networks within fully transparent, intact specimens, such as mouse brains [3,5,6,12,13,14,15,16,17,18,19]. Macro-LSFM utilizes a planar, micrometer-thin laser light sheet beam to illuminate expansive fields within large biological specimens at a cellular level of resolution, thereby facilitating the simultaneous acquisition of multiple optical sections and significantly accelerating the overall imaging process [13,15,16,17,19]. Importantly, this approach also minimizes the energy load on the biological specimen, minimizing the risks of photobleaching and phototoxic damage [7]. The elevated volumetric imaging speeds and inherent functionalities of Macro-LSFM prove particularly advantageous for live-imaging studies that demand both high spatiotemporal resolution and sustained durations of observation [7]. These capabilities open doors to the comprehensive imaging of entire fixed mammalian brains [7,8,20,21]. Of particular significance, our Macro-LSFM analysis results uncovered the presence of diffuse amyloid plaques (poorly marginated aggregates of Aβ peptide devoid of a central fibrillar core surrounded by dystrophic neurites and activated glial cells) that are not accessible with standard amyloid PET/CT modalities. Most amyloid PET/CT tracers are engineered for high affinity to fibrillar forms of Aβ plaques but exhibit reduced sensitivity for detecting diffuse plaques due to a confluence of factors: technical limitations such as suboptimal spatial resolution and poor signal-to-noise ratio; biological variability; and specific characteristics of diffuse plaques, including their lower density, absence of a dense fibrillar core, and lesser impact on surrounding tissue metabolism—collectively accounting for the modality’s diminished efficacy in identifying this particular form of amyloid deposit.

The development and refinement of animal models for Alzheimer’s disease (AD) have been cornerstone efforts in the scientific inquiry into the development and progression of the disease’s natural course, elucidating its etiology, mechanistic pathways, and associated risk factors [22]. These recent advances in the understanding of the pathophysiological mechanisms underlying AD have pointed to novel strategies for therapeutic interventions, thereby guiding the design of human clinical trials [22,23]. They provide robust frameworks for adequate testing paradigms for evaluating the effects of various therapeutic approaches [22,24]. The 5XFAD transgenic mouse model was developed in 2006, and these mice overexpress human amyloid precursor protein (*APP*) with three FAD mutations [the Swedish (*K670N*, *M671L*), Florida (*I716V*), and London (*V7171*) mutations] and human presenilin 1 (*PSEN1*) with two FAD mutations (*M146L* and *L286V*) [25]. The expression of both transgenes is regulated by neural-specific elements of the mouse Thy1 promoter to drive their overexpression specifically in brain neurons [26]. The 5xFAD transgenic mouse model has emerged as a critical instrument in the Alzheimer’s disease research community due to its unique genetic composition, which accelerates the timeline for the onset and progression of dementia symptoms. One of the most notable features of the 5xFAD model is its capacity for rapid accumulation of amyloid β pathology, which in turn expedites the bench-to-bedside transition of novel therapeutic compounds by allowing for immediate assessment of pharmacological interventions targeting plaque elimination or inhibition. The 5xFAD model mice display a high degree of congruency with the pathophysiological features observed in human AD patients, bolstering their translational relevance from mice to humans and thereby supporting research endeavors that directly feed into clinical trials [25,27,28]. According to the natural pathologic course of 5xFAD model mice published on AlzForum (https://www.alzforum.org/research-models/5xfad-b6sjl, accessed on 30 November 2023), these mice demonstrate the intraneuronal accumulation of Aβ42 peptides as early as 1.5 months, setting the initial stage for further pathological events [25]. By the age of two months, extracellular amyloid plaques begin to form in the subiculum and cortical layer V, simultaneously accompanied by the onset of astrogliosis and microgliosis [25]. This co-occurrence underlines the intricate relationship between amyloid pathology and neuroinflammation. Cognitive decline emerges between the ages of four and six months [25]. As the model ages to six months, amyloid plaques not only occupy the hippocampus and cortex but also extend to the thalamus, brainstem, and olfactory bulb [25]. By nine months, the mice exhibit significant neuronal loss [25]. This traditional pathologic trajectory course of 5xFAD model mice has reinforced the long-established dogma within the neurological field concerning the temporal relationship between neuroinflammation and neurodegeneration—specifically, the notion that an early phase of neuroinflammation precedes the subsequent late stage of neurodegeneration [29]. Considering that the 5xFAD mouse is commonly used (an estimated 10% of all AD studies utilizing animal models employ this strain (AlzPED; https://alzped.nia.nih.gov (last accessed on 30 November 2023)), the importance and impact of the data illuminating its natural pathological course, as notably highlighted on the Alzforum webpage where color-coded bands are used to represent distinct phenotypic characterizations, warrant considerable and indubitable emphasis. This framework significantly informs both our understanding of Alzheimer’s disease as well as the design and implementation of therapeutic approaches. However, the current information on the natural pathologic trajectory of 5xFAD published on Alzforum is mainly based on older research results, including a publication by Oakley et al. in 2006 [25]. The experimental methods primarily used in this study are limited to traditional experimental methodologies that predominantly encompass conventional immunoblotting, enzyme-linked immunosorbent assays (ELISAs), and immunohistochemical analyses conducted via light microscopy. Employing traditional evaluation methods, which are solely dependent on conventional staining methods and light microscopy, confines the scope of the investigation to pathology spot counting within exceedingly thin, two-dimensional frozen sections of localized subregions of the cerebral cortex [25]. These methodological constraints inevitably curtail the potential for a thorough, global understanding and morphological insight into the spatial distribution of the true three-dimensional amyloid and cellular ultrastructure volumes provided by comprehensive three-dimensional volumetric analysis, particularly with respect to the Z-axis. In contrast to traditional imaging techniques, which often necessitate the labor-intensive process of tissue sectioning, our state-of-the-art light sheet fluorescence microscopy (LSFM) imaging system incorporates the latest advancements in automated computational image analysis for three-dimensional volume rendering. This innovation enables comprehensive, cellular-level examination of intact transparent mouse brains, specifically focusing on the complex architecture and intricate cellular interaction among amyloid pathology and diverse cell types that constitute complex neuroinflammatory networks, including neurons, astrocytes, and microglia, a task virtually unachievable through 2D slices alone. 

Our emerging data generated by this cutting-edge technology challenges long-standing assumptions about the temporal dynamics between neuroinflammation and neurodegeneration. Accumulating bodies of recent evidence have highlighted the crucial role of reactive astrocytes as an integral player in AD pathology, providing new insights into their consequential implications. Canonical theories have traditionally asserted that the initial phases of neuroinflammation are antecedent to, and may even precipitate, the later phases of neurodegenerative decline. Several noteworthy studies, including in vivo animal-PET studies using transgenic AD mouse models and in vivo human PET (positron emission tomography) studies in families carrying autosomal dominant Alzheimer’s disease (ADAD) mutations, have collectively shown that reactive astrogliosis is markedly increased in the early stages of AD progression cascades, even before the deposition of Aβ and tau, and the onset of neurodegeneration [30]. In a longitudinal animal PET study with the amyloid plaque tracer [^11^C]AZD2184 and the astroglial tracer 11C-deuterium-L-deprenyl ([^11^C]DED), cortical and hippocampal [^11^C]DED PET binding was significantly higher at six months than at 8–15 months or 18–24 months in *APPswe* mice [31]. In the longitudinal, multi-tracer PET study conducted within the ADAD clinical cohort, the initially high, subsequently declining activity of astrocytosis, as measured by [^11^C]DED PET, in contrast to the steadily increasing amyloid burden during disease progression, indicates that active neuroinflammation plays a role in the early stages of the Alzheimer’s disease pathologic cascades, potentially up to 15–20 years prior to the clinical manifestation of symptoms [32,33]. However, in a series of in vivo [^11^C]DED PET and GFAP immuno-reactivity studies for clinical and preclinical animal subjects, researchers have observed a dual-phase bimodal astrocyte activation pattern at different stages of AD disease course [31,33,34,35,36,37]. In the context of the 5xFAD mouse model employed in our investigation, neuronal loss was documented to commence as early as six months of age. Thus, the findings observed in the 44-week-old (approximately 11-month) 5xFAD mice evaluated in our research are indicative of a late-stage neurodegenerative period, well past the onset of initial neuroinflammatory activity. Contrary to the long-standing finding that early-stage neuroinflammation precedes the subsequent later stage of neurodegeneration, our data reveal that active, late-stage neuroinflammation is still present in aged AD brains, even as they continue to show signs of ongoing neurodegeneration and significant amyloid accumulation. In line with an increased DED binding at the advanced/end stage of postmortem dementia sample [31,34,35,36], our observations correspond to the second, later phase of neuroinflammation, as suggested by the hypothetical model proposed by Kumar et al. [30]. The biological significance of the second wave, late-phase neuroinflammation observed in our data might represent a subgroup of resilient astrocytes that, while dormant in the early or preclinical stages, are nonetheless engaged in a protective role to avert complete neural collapse, and subsequently undergo a reactive transformation and tissue remodeling—culminating in scar formation—as a final effort to preserve existing neurological function in advanced stages of Alzheimer’s dementia [30]. Another possibility is that these astrocytes are a sub-population of disease-associated astrocytes, known as DAAs, characterized by high GFAP expression states that become reactive with aging [38,39]. In aged wild-type mice and postmortem human brains, disease-associated astrocytes, or DAAs, appear to undergo upregulation in the early stages of the disease, becoming increasingly prominent and abundant as the disease advances, and are frequently found in close proximity to amyloid-beta (Aβ) deposits [38,39]. 

Our study has several limitations. First, the age of our control *C57BL/6J* group does not perfectly match to the 44-week-old 5xFAD experimental group. Our initial choice of the 31-week-old *C57BL/6J* mice as a control group was based on the judgment that 31 weeks of age, represented a mature but not aged stage, appropriate for comparison with the older 5xFAD model. Because aging is an important confounding factor in AD pathology, incorporating both young (6-month-old) and old (10-month-old) mice from both the 5xFAD and *C57BL/6J* strains would enable a more nuanced analysis of the interaction between aging and the Alzheimer’s disease (AD) genotype in neuroinflammatory processes. A second limitation arises from the technical challenges associated with the use of multiple antibodies simultaneously, which can lead to reduced antibody penetration, extended experimental timelines, and compromised imaging quality. Therefore, our ability to expand our immunolabeling to include a broader spectrum of neurotoxic and neuroprotective markers for astrocytes or microglia, in addition to the established green (488 nm), red (561 nm), and purple (647 nm) channels, has been constrained. In light of the biological complexity of astrocyte heterogeneity [40,41,42], whether the role of late-phase neuroinflammation in our data is implicated in either neuroprotective or detrimental signaling pathways remains an unresolved question. Third, a further limitation of our research is the absence of correlative data linking the three-dimensional distribution of neuropathological features to cognitive and structural brain MRI evaluations, an element vital for comprehending the clinical ramifications of late-stage neuroinflammation on cognitive impairment and the aggravation of structural neurodegeneration. Subsequent investigations would benefit from a longitudinal approach involving both young and aged transgenic models, which would elucidate the temporal evolution of neuroinflammatory and neurodegenerative processes and their cognitive sequelae. Finally, the current analytical approach utilized in our study, employing Imaris software (Version 7.2.3, Bitplane AG, Zurich, Switzerland), is designed for global brain analysis through automated thresholding. Despite providing substantial whole-brain insights, this method lacks the advanced processing capabilities needed for fully automated, region-specific brain mapping in accordance with an integrated mouse brain atlas. Such targeted analyses are critical for evaluating specific brain areas like the hippocampus and cortex, which are integral to cognitive function and particularly susceptible to the pathophysiology of AD.

Utilizing advanced macro-laser light-sheet microscopy and state-of-the-art tissue-clearing techniques, our comprehensive investigation into the three-dimensional spatial distribution of key neuropathological AD markers in the chemically cleared brains of 44-week-old 5xFAD mice revealed that active neuroinflammation persists even in the late-stage neurodegeneration phase in the aged AD brain, marked by substantial amyloid deposition, challenging the traditional canonical sequence of early neuroinflammation leading to late-stage neurodegeneration. Given the widespread utility of the 5xFAD model in dementia research, our data, which challenges the conventional understanding of the natural pathological progression in 5xFAD, holds profound implications for the contemporary landscape of Alzheimer’s disease research, influencing the design and implementation of anti-neuroinflammatory therapeutic strategies.

## 4. Materials and Methods

### 4.1. Animal Model

For the current investigation, we assembled an experimental cohort of ten 44-week-old female B6SJL-Tg (APPSwFlLon, PSEN1*M146L*L286V) 6799Vas/Mmjax mice, commonly referred to as the 5xFAD model, that were obtained from The Jackson Laboratory, Bar Harbor, ME, USA. Our selection of the female 5xFAD mouse model for this study is predicated on the robust findings of Poon et al. [43], which demonstrate sex-specific variances in AD pathology, particularly, an earlier onset and hastened progression in females. Additionally, seven 31-week-old female *C57BL/6J* mice were used as comparative mature controls. The 5xFAD mouse model, enriched with five familial Alzheimer’s disease (AD) mutations in the *APP* and *PSEN1* genes, serves as an instrumental platform for unraveling the temporal and spatial complexities of Alzheimer’s disease pathology [25]. Beginning with the intraneuronal accretion of *Aβ42* peptides as early as 1.5 months, the model sets the preliminary stage for a sequence of progressive pathological phenomena [25]. Around the age of 2 months, extracellular amyloid plaques begin to be deposited in the subiculum and cortical layer V [25]. Concomitantly, astrogliosis and microgliosis initiate, developing in parallel to plaque formation and thus illuminating the closely orchestrated interaction between amyloid pathology and neuroinflammation [25]. Cognitive decline emerges between the ages of 4 and 6 months [25]. As the model mice age to 6 months, amyloid plaques not only occupy the hippocampus and cortex but also extend to the thalamus, brainstem, and olfactory bulb [25]. By 9 months, the mice exhibit significant neuronal loss [25]. All mice were housed in a temperature-regulated environment (22 ± 2 °C, 55 ± 10% relative humidity) with a 12-h/12-h light/dark cycle, ad libitum access to standard rodent feed and water, and accommodation in standard polycarbonate cages with autoclaved wood shavings and enrichment materials. The mice were allowed a one-week acclimatization period prior to any experimental procedures. All animal experiments were conducted following approval from the Dankook Institutional Animal Care and Use Committee, adhering to the respective guidelines and regulations (DKU-22-080).

### 4.2. Hydrophilic Clearing Process of Brain Samples

In compliance with protocols established by the Institutional Animal Care and Use Committee (IACUC), the mice were euthanized using isoflurane. Subsequently, transcranial perfusion employing 1× phosphate-buffered saline (PBS) was performed to effectively eliminate blood from the cerebral vasculature. Tissues were then fixed in a 4% paraformaldehyde (PFA) solution dissolved in PBS. The subsequent steps involved careful dissection of the brain, and the dissected samples were then subjected to overnight postfixation at 4 °C using 4% PFA. Further dissection targeted a 1 mm sagittal section, specifically focusing on the CA1 hippocampal region, with coordinates relative to bregma as follows: −2 mm anterior/posterior, ±1.8 mm lateral/medial, and −1.5 mm dorsal/ventral. Tissue clearing was achieved using a hydrophilic Binaree Tissue Clearing Solution Kit (HRTI-001, Binaree, Daegu, Korea). PFA was washed off with 1× PBS thrice at 4 °C, and each wash lasted for 20 min. The samples were then submerged in 10 mL of starting solution, followed by incubation at 37 °C for 48 h while shaking at 50 rpm. Next, the solution in each tube was replaced with 10 mL of Tissue Clearing Solution A, and the incubation process was repeated. Each tube was then rinsed four times with distilled water at 4 °C, and each wash lasted for 30 min. The solution was subsequently substituted with 10 mL of Tissue Clearing Solution B, and the incubation process was repeated once more. Finally, a 48-h permeabilization process was undertaken using a solution comprising 0.2% Triton X-100 and 10% dimethyl sulfoxide (DMSO) in 0.1× PBS. This step was critical for facilitating subsequent analyses requiring membrane penetration.

### 4.3. Volume Immunostaining for β-Amyloid Plaques, Astrocytes, and Microglia

To visualize β-amyloid plaques, specimens were treated with thioflavin S, which emits at 488 nm. Astrocytes were identified using an anti-mouse ACSA-2 primary antibody from Miltenyi Biotec with a 647 nm emission spectrum. Concurrently, microglia were labeled with an anti-mouse CD11b monoclonal direct primary antibody obtained from eBioscience™ (San Diego, CA, USA), featuring a 561 nm emission peak. The applied protocol necessitated a five-day incubation at 37 °C under sustained agitation. Following this phase, samples were extensively rinsed six times with 0.1× phosphate-buffered saline (PBS) before being subjected to secondary antibody incubation. This secondary stage also employed an anti-mouse ACSA-2 antibody from Miltenyi Biotec and lasted for an additional five days at 37 °C, with ongoing agitation. For antibody dilution, a buffer containing 0.2% Tween 20 and 10% dimethyl sulfoxide (DMSO) in 0.1× PBS was used. β-Amyloid plaques were stained using a 1% thioflavin S solution dissolved in 50% ethanol. Samples were incubated at 25 °C, shaken at 50 rpm for 15 min, and subsequently washed three times with 1× PBS at 4 °C, with each wash lasting 20 min. To match the high refractive index (RI) of the tissue and render tissues transparent, the samples were incubated in 6 mL of Mounting and Storage Solution (SHMS-060, Binaree, Daegu, Korea) at 37 °C for 48 h while shaking at 50 rpm. Mounted samples were stored in the dark at room temperature until light sheet fluorescence microscopy (LSFM) imaging.

### 4.4. Macrolaser Light-Sheet Illuminating Microscopy (Macro-LSFM) Imaging Acquisition and Imaris Software-Based Automated Quantitative Analysis of 3D Volumetric Surface Model Generation

High-resolution Macro-LSFM (light sheet fluorescence microscopy) imaging of ex vivo cerebrum specimens was conducted on a Zeiss Lightsheet Z.1 microscopy system (Carl Zeiss Meditec, Inc., Oberkochen, Germany). For optical acquisition, a quintuple magnification objective lens (NA = 0.16) was used, coupled with a zoom factor of 0.75×. The LSFM apparatus was calibrated with a triad of excitation laser wavelengths specifically chosen for targeted cellular markers: 647 nm for astrocytes, 555 nm for microglia, and 488 nm for the detection of β-amyloid aggregation. A single-illumination mode was deployed to enhance both the signal-to-noise ratio and overall image clarity. Following the acquisition, raw data were transcribed into CZI (Carl Zeiss Image) file format for subsequent analytical processing. Quantitative image evaluation was executed via Imaris software (Version 7.2.3, Bitplane AG, Zurich, Switzerland). Native CZI files were transmuted into an Imaris-compatible data schema for further analysis. As described in the previous literature [44], the dimensional attributes of the three-dimensional volumetric data (encompassing length, width, and height) were each downscaled to 25% of their original extents. This transformation concomitantly resulted in a reduction in voxel dimensions, thus optimizing computational efficiency during image analysis. For each of the selected laser wavelengths (647 nm for astrocytes, 555 nm for microglia, and 488 nm for β-amyloid), a computational surface model was generated via the Surface Creation Wizard integrated within the Imaris software environment. The minimum discernible feature size was prespecified at 4.0 μm, which corresponds to the smallest detected diameter of the targeted cellular markers within the slice-mode observations. To segregate the true signal from the extraneous background, local contrast enhancement was employed for background subtraction. An automated thresholding approach was initially utilized to define the largest permissible feature size in the extraneous background, which was subsequently refined through manual adjustments. This ensured that the constructed surfaces authentically replicated the morphological attributes of the cellular markers while simultaneously minimizing the incorporation of aberrant noise. Statistical validation was carried out by applying a 95% confidence interval to the voxel dimensions, thereby accurately determining the volumetric characteristics of the surface models. Volumetric data extraction was facilitated through the use of the “Statistics” functionality located under the “Surpass” tab within the Imaris interface. The “Volume” metric was specifically selected from the assortment of available measurement parameters.

### 4.5. Statistical Analysis

Data are shown as the median [minimum–maximum] unless otherwise stated. A value of *p* < 0.05 was considered to be statistically significant. The Mann–Whitney test was used to determine the differences in amyloid, astrocyte, and microglia volume between AD and control mouse models. Linear regression analyses were used to evaluate the association between the amyloid volume of the brain and that of the eyeball. All statistical analyses were performed with MedCalc^®^ Statistical Software version 20.015 (MedCalc Software Ltd., Ostend, Belgium).

## Figures and Tables

**Table 1:** Comparison of the total surface volume of amyloid, astrocytes, and microglia between the Alzheimer’s disease (AD, *n* = 10) and control mouse (*n* = 7) groups.

Volume	AD Mice	Control Mice	*p*
Amyloid (brain), µm^3^	898,634,368 [383,355,488–1,324,986,752]	33,320,178 [11,156,785–65,390,988]	0.0006 *
Astrocyte (brain), µm^3^	59,064,360 [27,815,500–222,619,280]	20,272,722 [9,317,288–27,223,352]	0.0047 *
Microglia (brain), µm^3^	51,210,100 [15,309,118–135,532,144]	23,461,593 [14,499,170–27,924,110]	0.0162 *

* *p <* 0.05 was considered statistically significant.

## Data Availability

Data are contained within the article.

## Supplementary Materials

The supporting information can be downloaded at: https://www.mdpi.com/article/10.3390/ijms242317058/s1.
